# ‘The Lost Peace’: Evidencing the Syndemic Relationship between Neglected Tropical Diseases and Mental Distress in Liberia

**DOI:** 10.3390/tropicalmed9080183

**Published:** 2024-08-17

**Authors:** Rosalind McCollum, Carrie Barrett, Georgina Zawolo, Rachel Johnstone, Tiawanlyn G. Godwin-Akpan, Hannah Berrian, Shahreen Chowdhury, Jerry Kollie, Karsor Kollie, Emerson Rogers, Colleen Parker, Maneesh Phillip, Lucas Sempe, Maaike Seekles, John Solunta Smith, Wede Seekey, Anna Wickenden, Zeela Zaizay, Sally Theobald, Laura Dean

**Affiliations:** 1 Department of International Public Health, Liverpool School of Tropical Medicine, Liverpool L3 5QA, UK; 2 Pacific Institute for Research and Evaluation, University of Liberia, Monrovia 1000, Liberia; 3 Programs Department, American Leprosy Mission, 27 Jungle Road, East Legon, Accra GA-374-5385, Ghana; 4 Department of Health Services, Ministry of Health-Liberia, Congo Town Back Road, Monrovia 1000, Liberia; 5 International Programs Department, Effect Hope, 200-90 Allstate Pkwy, Markham, ON L3R 6H3, Canada; 6 The Institute for Global Health and Development Division, Queen Margaret University, Edinburgh EH21 6UU, UK; 7 Action Transforming Lives, Congo Town Backroad, Monrovia 1000, Liberia

**Keywords:** syndemics, neglected tropical diseases, Liberia, mental health, depression, anxiety, disability

## Abstract

Neglected Tropical Diseases (NTDs) are a group of chronic infectious diseases of poverty affecting over one billion people globally. Intersections of NTDs, disability, and mental ill-health are increasingly evidenced but are rarely studied from a mixed-methods perspective. Here, we advance syndemic understandings by further assessing and contextualising the syndemic relationship between NTDs (particularly their associated disability) and mental distress in Liberia. Participatory qualitative methods, including body mapping (56 participants), social mapping (28 participants), and in-depth interviews (12) provided space for persons affected by NTDs to narrate their experiences. Simultaneously, 201 surveys explored experiences of common mental health conditions among persons affected by skin NTDs. An intersectionality approach was applied within the analysis for both qualitative and quantitative methods informed by Meyer’s minority stress model, adapted for NTDs. Qualitative data was analysed thematically and gender-disaggregated, univariable and multivariable analyses were applied to survey data for the outcome measures depression (PHQ-9) and anxiety (GAD-7). Disability was associated with higher levels of depression and anxiety (*p* < 0.001). An interaction between disability and being a women increased incidence risk ratio of depression (*p* < 0.001). In alignment with qualitative findings, persons affected experienced additional generalised (financial concerns), external (experience of stigma) and internal (experience of pain and physical symptoms) minority stressors, to varying degrees, which contributed towards their mental distress, and mental health conditions. These findings were used to co-develop a syndemic-informed person-centred health system response to address the suffering associated with NTDs and mental distress, including a focus on strengthening relationships between formal and informal community health actors and the broader health system.

## 1. Introduction

Neglected Tropical Diseases (NTDs) are a group of chronic infectious diseases of poverty that affect more than one billion people, primarily from low- and middle-income countries (LMICs) [1,2]. NTDs, particularly those that affect the skin, lymphatic filariasis (LF), leprosy, Buruli ulcer, Yaws and onchocerciasis, present significant pain and physical disfigurement; stigma and discrimination are also common experiences [3]. LF and onchocerciasis are vector borne diseases caused by filarial nematodes, LF presents most commonly as hydrocoele and lymphoedema, and onchocerciasis can cause disfiguring skin conditions and blindness [4]. An estimated 36 million people are living with LF symptoms, and 14.6 million people living with skin disease and 1.15 million with blindness as a result of onchocerciasis [5,6,7]. Yaws is caused by a bacterium and characterised by papillomas (noncancerous lumps) and ulcers, with over 80,000 new cases reported each year [8]. Leprosy affects the skin and the peripheral nerves and Buruli ulcer leads to ulceration and skin loss, both are caused by a different species of mycobacterium, and transmission routes are not fully understood [9,10,11]. Over 200,000 new leprosy cases are reported each year within 120 countries [9].

Mental distress and mental health conditions (depression and anxiety) amongst people affected by NTDs are significantly higher than in the general population [3,12,13,14,15,16,17,18,19]. The link between NTDs and common mental health conditions (depression, anxiety and suicidal ideation) is increasingly documented through epidemiological clustering studies [16,20,21,22,23,24,25,26]. A recent review evidences three multi-directional pathways that are shaping epidemiological clustering, namely: (1) people experiencing mental distress or mental health conditions are more at risk to NTDs, and people with NTDs are more at risk to mental distress; (2) people with NTDs experience increased levels of stigma and discrimination leading to higher levels of mental distress; and (3) people with mental health conditions experience high levels of stigma and discrimination heightening vulnerability to NTDs due to delayed care seeking and the poverty-disability nexus [15]. Stigma is often identified as a catalyst of such epidemiological clustering, yet limited research in the field of mental health, stigma and NTDs has taken an equity lens, guided by gendered and intersectionality theory, to understand the drivers of these burdens [3,27]. Thus, understanding broader meso and macro social and structural inequalities driving these multi-directional pathways becomes essential to ensure appropriate person-centred responses for people affected by NTDs and mental health conditions [3,15]. The application of syndemic theory, that considers the biosocial relationships that drive ‘synergistically related’ epidemics, becomes critical and essential in shaping these understandings [28]. 

Person-centred responses to NTDs and mental health conditions require a re-orientation of health systems, so that holistic understandings of health (i.e., those that extend beyond the biomedical) are championed. Adjusting care models in this way requires a prioritisation of primary health care interventions that are co-produced with persons affected by NTDs and communities who become active change agents promoting good health and wellbeing throughout the life-course [26,27]. Yet, evidence on ‘what works’ to address the synergistically related epidemics of NTDs and mental (ill-) health is sparse, and where intervention(s) do exist they have largely been top-down with limited integration within national health systems [29]. Further contextualising the syndemic relationship between NTDs and mental distress, including moving beyond stigma, to think about additional underlying social and structural inequities, becomes imperative to support evidence based, demand driven systems reform. People affected by NTDs and mental health conditions are critical assets in this process, and co-production should be a central value in promoting positive shifts toward normative frameworks of person-centred care [17,27]. 

### 1.1. The Context of Liberia

Liberia has experienced protracted drivers of fragility in the past 3 decades, including civil wars (1989–1996, 1999–2003), Ebola virus disease (EVD) epidemic (2013–2016) and the COVID-19 pandemic [30]. Liberia’s unique political history, shaped by the post-slavery, post-colonial experience, where disunity between ‘settlers’ (freed American slaves) and indigenous peoples contributed to protracted unrest and fragility [31]. Subsequent economic mismanagement shapes the ongoing high levels of poverty (with 50.9% of Liberians living below the national poverty line [32]); low levels of education and a weakened health system [31]. These factors contribute to a reliance on traditional medicine by many Liberians. The health system was further devastated by the deaths of 192 health workers responding to the EVD epidemic [33]. These macro-level determinants predispose people to the chronic effects of NTDs and mental distress [31]. Consequently, experiences of mental ill-health are common, although evidence is limited [30], estimates indicate 40% of the population meeting the criteria for major depressive disorder (albeit based on data from 2008) [34]. Infectious diseases of poverty, including NTDs, are frequently diagnosed late, coupled with poor management of NTDs due to limited systems capacity, causes increased morbidity and mortality [3]. Dean (2022), characterise the relationship between mental distress and NTDs (particularly their associated chronic morbidity/disability e.g., lymphoedema) in Liberia as a syndemic driven by three key factors: (1) ongoing structural violence in Liberia pre-disposes people and communities to the chronic effects of NTDs as well as other generalised stressors (e.g., poverty); (2) people affected by NTDs and or mental health conditions face additional stressors, largely related to stigma and discrimination; and (3) the impact of general stressors, and NTD/mental health related stressors synergistically interact with identity based characteristics, including gender and age to shape individual experiences of syndemic suffering [3]. However, within the analysis presented by Dean (2022), an epidemiological evidence gap remains due to the lack of quantitative studies considering the epidemiological clustering of mental health conditions and NTDs in this context. The syndemic is theorised through the application and adaptation of Mendenhall’s (2017) model of syndemic approaches to health, with stigma highlighted as a critical catalyst through the adaptation and application of Meyer’s (2003) minority stress model [28,35]. 

Within this study, we draw on mixed-methods approaches to further contextualise the syndemic relationship between NTDs (and their associated disability) and mental distress (depression, anxiety) in Liberia. The biological interaction between the NTDs of focus and mental health conditions has not been explicitly explored, with the exception of onchocerciasis which is increasingly linked to neurodevelopment disorders [36]. Whilst no research has specially looked at the biological relationship between NTDs and mental distress, some research has highlighted the vicious cycle of stress-induced inflammatory events related to other skin conditions, potentially leading to worsening of symptoms [37]. Physical pain, disability stage, and duration of illness leads to poor mental health outcomes among persons affected by NTDs; poor mental health delays health seeking, negatively impacting physical health; NTD related medications may impair mood and cause anxiety, agitation or psychosis; and some NTDs directly affect the brain, leading to mental and neurological consequences [3,13,16,17]. Although, this hasn’t been investigated extensively for NTDs, by advancing the Consequently, within this study, we focus on the social and structural mechanisms through which NTDs and mental health conditions synergistically interact and exacerbate health inequalities, as a stigma syndemic. However, we do recognise that further study of the biological interactions between NTDs and mental distress is needed to advance the syndemic argument [38].

This study was completed to support the development of person-centred approaches to skin NTDs, including the integration of mental health services in Liberia, through the REDRESS programme. REDRESS is a five-year, multi-disciplinary research consortium established at the request of the Ministry of Health, Liberia to identify effective strategies to detect, refer, treat and support people living with NTDs that are acceptable, affordable and sustainable especially amongst the most vulnerable [39]. REDRESSS’ underpinning methodology is participatory action research to support the co-designing and testing of innovative health systems interventions. In line with participatory health research values and principles [40], this study prioritises the views and experiences of people affected by NTDs, including them as co-researchers within study design, delivery and analysis. 

### 1.2. Contextual Framework: Meyer’s Minority Stress Model 

**Figure 1:**
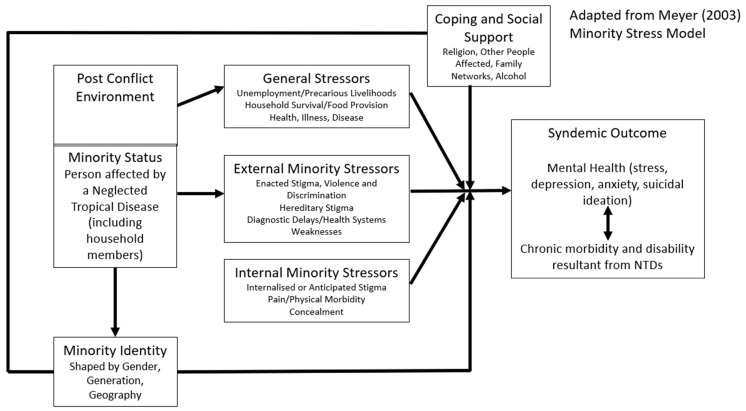
Minority stress model for NTDs and mental distress, reprinted with permission from Dean (2022) [3] which is an adapted from Meyer (2003) [35].

Meyer’s minority stress theory argues that minority individuals experience psychosocial stress as a consequence of stigma and discrimination that is experienced as direct result of a minority identity [35]. Minority stress theory was originally documented and theorised in relation to the experiences of sexual minorities, largely in the United States [35]. However, Dean (2022) have adapted this model in relation to NTDs in Liberia based on narrative accounts of people affected [3]. In their adaptation and application of Meyer’s model, Dean (2022) (see Figure 1) hypothesise that the disadvantage associated with being diagnosed with an NTD presents a minority status. This minority status is thought to exacerbate ‘general stressors’ for the majority of individuals and communities in Liberia which exist as a consequence of ongoing structural violence due to the post conflict environment, coupled with additional periods of fragility, as a consequence of multiple disease outbreaks (including Ebola, COVID-19). External and internal minority stressors associated with being affected by an NTD then become added stressors that can contribute to poorer, synergistically related health outcomes, specifically, mental distress (depression, anxiety, suicidal ideation) and morbidity/disability. Intersecting axes of inequity, such as gender, age and geography (minority identities) are described as having an interactional relationship with these stressors shaping embodiment/internalisation of oppression and nuanced experiences of the syndemic. 

## 2. Methodology

### 2.1. Study Context and Sites 

We used mixed-method approaches to further interrogate the syndemic relationship between skin NTDs of priority (yaws, Buruli ulcer, LF, onchocerciasis, leprosy) and mental distress (anxiety, depression and suicidal ideation) for persons affected in Liberia. Participatory, qualitative and quantitative data was collected from persons affected by skin NTDs. Drawing on participatory health research principles [40], we involved co-researchers as part of the research team, including community health workers and affected people. Co-researchers received training in research methods, safeguarding and ethics, and took part in data collection, analysis and validation. 

The REDRESS study was implemented within three counties in Liberia: Grand Gedeh, Lofa and Margabi (subsequently referred to as the ‘REDRESS counties’). These counties were purposively selected in collaboration with the Ministry of Health, Liberia to ensure maximum variation in: (1) the service delivery context of the integrated case management of skin NTDs; (2) endemicity (for all skin NTDs of focus); (3) geography (peri-urban and rural); and (4) socio-demographics (e.g., ethnicity, literacy).

### 2.2. Survey Data Collection

We completed a cross-sectional survey in the three ‘REDRESS counties’ (Grand Gedeh, Lofa, Margabi) and one comparator (for the main REDRESS evaluation) county Grand Kru between October 2022 and January 2023 to ascertain the mental health status of adults (>18 years) affected by one of the five endemic skin-NTDs. Grand Kru was purposively selected as a comparator due to similarities in disease endemicity, geography and health service delivery to the ‘REDRESS counties’. This cross-sectional survey was part of the wider REDRESS study to evaluate subsequent interventions. Consequently, the sample size of 75 men and 75 women was derived to observe changes in depression scores (PHQ-9) pre- and post-REDRESS interventions and comparisons between gender.

All survey data was collected using REDCAP software (version 13.7.6) and uploaded via electronic tablets (Samsung Galaxy Tab A7 Lite, Monrovia, Liberia). Data were downloaded as a CSV file and imported onto R programme (version 4.3.1) for descriptive and statistical analysis.

### 2.3. Survey Information

Participants were identified by co-researchers from facility registers, in partnership with trained community health assistants and verified (as living with an NTD) by county health NTD focal persons. Survey questions focused on the participants socio-demographics (gender, age, education level, county), disease (NTD), common mental health conditions (depression and anxiety), stigma and disability scores. Education level was self-reported and described in three categories: no education (i.e., never attended school); attending primary education (i.e., elementary (Grades 1–6)); and attended above primary level education (Grades 7+). 

#### Outcome Measures

Depression and anxiety were assessed using the 9-item Patient Health Questionnaire (PHQ-9) and Generalised Anxiety Disorder 7-item (GAD-7) questionnaire, respectively [41,42]. Stigma was assessed using the Stigma Assessment and Reduction of Impact (SARI) scale and disability was assessed using the World Health Organization (WHO) Disability Assessment Schedule 2.0 (WHODAS 2.0) [43,44]. A Likert scoring system was used to collect questionnaire answers which ranged from 0–27 for depression (PHQ-9), 0–21 for anxiety (GAD-7), 0–100 for stigma (SARI) and 0–4 for disability (WHODAS). The PHQ-9 tool has widely defined cut-off score categories, no depression (0–4), mild depression (5–9), moderate depression (10–14), moderately severe depression (15–19) and severe depression (20–27), previously used in Liberia [45,46]. Similarly, GAD-7 categorised no anxiety (0–5), mild anxiety (5–9), moderate anxiety (10–14), severe anxiety (15–21). There are no validated cut-off scores for the stigma SARI score and degrees of disability and so this score was used as a count variable. WHODAS score for disability defined as categories, no disability (0), mild disability (1), moderate disability (2), severe disability (3) and extreme disability (4) [47].

### 2.4. Qualitative Data Collection

A mixture of qualitative (in depth interviews (12) and participatory methods (body mapping (56 participants), social mapping (28 participants)) were used in order to help understand the experiences, meanings and views of persons affected (Pope and Mays 1995). Qualitative data collection took place across five counties in Liberia, the three ‘REDRESS counties’, plus an additional two purposively selected counties (Bong and Nimba) between June 2021 and July 2022. Bong and Nimba were purposively selected due to the high prevalence of endemic skin NTDs. Participants for the in depth interviews (IDIs), body mapping, and social mapping were purposively selected from facility records to support maximum variation in gender, age and disease condition.

In depth interviews explored more about individual experiences with their condition, including both physical and mental wellbeing, seeking care, health beliefs, stigma, and participation within community life. Body mapping was either completed during a group discussion (Grand Gedeh, Lofa, and Margibi) (8 groups) or on a one-to-one basis (Bong and Nimba) (21 participants). Participants were invited to draw a map of themselves and to add to this symbols and images relating to their experiences with their condition, including mental and physical wellbeing. Social mapping was conducted in a group setting and participants were invited to draw out their community identifying key locations for social gatherings within the community, e.g., church or mosque, water pump. This map was then used as a prompt for discussion about their participation within the community. All interviews were audio recorded, transcribed verbatim and stored to SharePoint. A selection of approximately 10% were randomly selected, with the transcript cross-checked against the audio for quality assurance purposes. 

### 2.5. Data Analysis

All data analysis was guided by the main dimensions within Meyer’s (2003) minority stress model: general stressors, external minority stressors, internal minority stressors, coping and social support (Meyer 2003). We also considered minority identity by drawing on intersectionality theory to consider how experiences are mediated by social and structural processes to create unique experiences for individuals based on their personal characteristics through the application of intra-categorical analysis (e.g., how are the experiences of men and women living with NTDs aligned/different) [48,49]. 

#### 2.5.1. Qualitative—Data Analysis 

For the qualitative data, thematic framework analysis was carried out to help classify and organise the data according to the main themes, concepts and categories emerging from the data [50]. Categories, guided by the model, were deductively identified, although space was provided to identify emerging issues inductively to ensure an iterative approach to analysis. As a research team (including co-researchers) we familiarised ourselves with the data in the transcripts before then jointly discussing the main themes within the transcripts which were subsequently developed into the coding framework. The coding framework was applied to data using Nvivo 12 to help manage data as part of analysis. After coding of the data, charts were developed, with narratives developed which included comparisons drawn based on gender, county and disease condition. 

#### 2.5.2. Quantitative—Data Analysis 

For the survey data, two sets of analysis were conducted each with two arms and guided by Meyer’s minority stress model and emerging issues identified within the qualitative data: (1) gender-disaggregated descriptive analysis with (i) socio-demographics and (ii) mental health (depression, anxiety and suicide ideation (iii), stigma and disability; (2) risk factor analysis: (i) univariable and (ii) multivariable analysis. Univariable analysis was performed on candidate risk factors: gender, age, diagnosed NTD, county and education level, stigma and disability. Relative-risk ratios (RRs), 95% confidence intervals (CIs) were plotted, and *p*-values were calculated and reported for all candidate risk factors. The internal reliability of the PHQ-9 and GAD-7 was derived the Cronbach’s α calculation. 

Gender-disaggregated descriptive analysis was used to understand differences between genders. The overall prevalence and 95% CI for depression, suicide ideation, anxiety, stigma and disability, reported and t-tests were performed. Chi-squared tests were performed to understand the relationship between gender and categories (i.e., mild, moderate, severe etc) for depression, anxiety and disability. 

Multi-variable analysis was conducted to understand key risk factors interactions and associations with depression and anxiety and a model selection process was performed. Gender-disaggregated and univariable analysis identified candidate risk factors to be carried forward to multi-variable modelling stage, by screening for statistical significance (*p*-value < 0.05). Multi-variable models were performed, firstly including all identified candidate risk factors and secondly investigating candidate risk factor interactions. Multi-variable modelling and quantitative findings identified key risk factors associated with depression and anxiety which informed the final models reported in this paper, which included with and without interactions between identified key risk factors. Stigma was excluded from final models due to missing data entries, and anxiety was excluded due to the co-occurrence of depression and anxiety [51,52].

### 2.6. Ethical Considerations

All researchers and co-researchers took part in training about safeguarding and considerations of power dynamics prior to data collection. The study received ethical approvals from the Liverpool School of Tropical Medicine Research Ethic Committee, United Kingdom (Protocol ID 20-040) and University of Liberia Pacific Institute for Research and Evaluation (UL-PIRE)’s Institutional Review Board, Liberia (Protocol ID 20-09-233) in March and April 2020, respectively. All participants were provided with information about the study and interview. Written informed consent was obtained from all participants.

### 2.7. Findings

A total of 201 participants were included in the survey (Table S1), across Grand Gedeh (N= 41 [20.4%]), Lofa (N = 72 [35.8]), Margibi (N = 34 [16.9%]) and Grand Kru (N = 53 [26.4%]). More than two-thirds of the participants were male (N = 125 [62.2%]) and the remaining female (N = 76 [37.8%]), with the total average age 46 years (range from 18 to 87) standard deviation (SD) of 16.0). Participants presented with five skin-NTDs; Buruli ulcer (N = 70 [34.8%]), LF-related hydrocoele (N = 49 [24.4%]), LF-related lymphoedema (N = 54 [26.9%]), leprosy (N = 21 [10.5%]), Yaws (N = 6 [3.0]), onchocerciasis (N = 1 [0.5%]). The majority of participants did attend primary education or above, with (N = 79 [39.3%]) attending higher than primary education, (N = 44 [21.9%]) only attending primary school education and (N = 77 [38.3%]) not attending education.

A total of 96 participants took part in qualitative and or participatory data collection activities (Table S2). 

### 2.8. Syndemic Outcomes

Our mixed-methods evidence is indicative of a syndemic relationship between NTDs and mental health, specifically depression, anxiety and suicidal ideation in Liberia, as described in Table 1. Of 201 participants surveyed, 47.8% (95% CI 40.7 to 54.9) reported moderate depressive symptoms or above (PHQ-9 ≥ 10). For anxiety, 30.8% (95% CI 24.5 to 54.9) reported moderate or severe symptoms (GAD-7 ≥ 10). The internal reliability for both the PHQ-9 and GAD-7 was assessed as excellent, with Cronbach’s α of 0.9 and 0.88, respectively. 

Comparison of gender means for depression (t(164) = 3.92, *p* < 0.001) and anxiety (t(164) = 3.94, *p* < 0.001), showed statically significantly higher scores in women in comparison to males. Additionally, women had statistically significantly higher disability scores (t(158) = 3.64, *p* < 0.001). Of the 201 baseline surveys, 128 had available stigma SARI scores, used for statistical analysis. No statistically significant difference was found between gender in overall stigma, experienced (enacted) and anticipated stigma SARI scores, and internalised stigma domain scores (t(90.9) = 2.54, *p* = 0.013) were statistically significantly higher amongst women.

**Figure 2:**
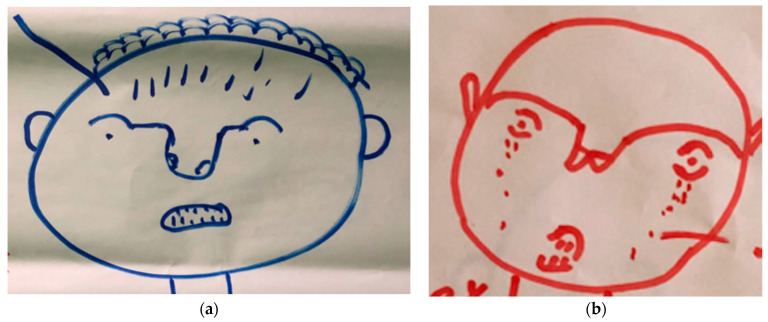
(**a**) Bodymap with male participants, Margibi. (**b**) Bodymap with male participant with BU, Lofa.

Qualitatively, irrespective of age or gender, respondents described feelings related to their mental wellbeing as ‘feeling bad’, ‘thinking too much’, ‘feeling low’, ‘very bad’ or ‘very sad and crying’. Anxiety was indicated within one body map as small lines inside the head (Figure 2a), with sadness and loneliness indicated with tears or crying (Figure 2b).

Women more frequently described feeling hopeless about their situation. A minority of respondents described thoughts of self-harm or suicidal ideation, as also apparent within the quantitative data with 47.8% (n = 96) of participants identifying as having thoughts of self-harm or suicide within the last two weeks. Suicidal ideation was also described qualitatively. A male participant, living with lymphoedema further articulated the connection between their mental and physical wellbeing, highlighting the detrimental impact of stress on them physically.

*“Sometime[s] when I go to bed, I can’t sleep. I will be worrying, thinking, thinking, thinking, until I understand. So, people just advise me now I need not to worry now... So, I need not to be worrying too much. Because the more I worry, that the more my body will be draining.”*

***Bodymapping male person affected by lymphoedema, 46 years old, Margibi***

### 2.9. Risk Factor Analysis

**Figure 3:**
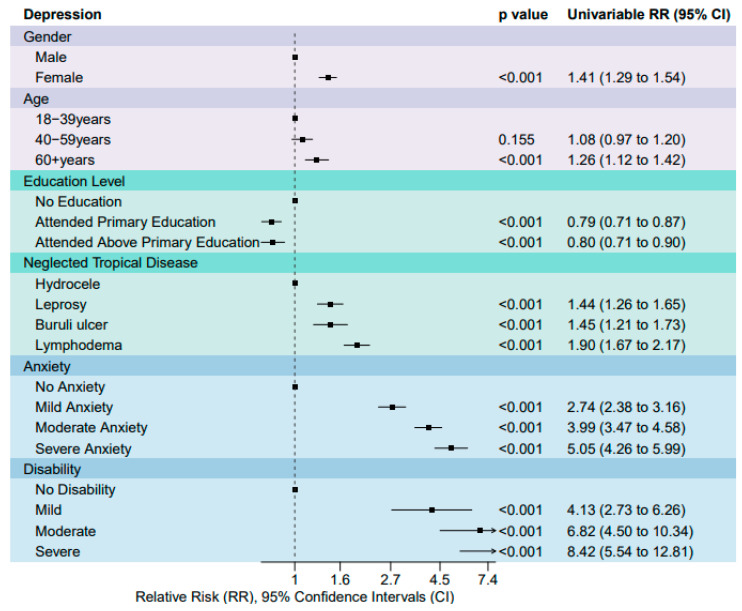
Relative-risk Ratios (RR) and 95% CIs for depression derived from univariable generalised linear regression Poisson models. RR above 1 (dotted line) shows a greater association with depression and RR below 1 means a lesser association in comparison to the first item listed for each variable.

For depression, Figure 3 shows estimated RRs, 95% CIs and *p*-values for univariable generalised linear regression Poisson analysis with all candidate risk factors. Participants that were female (RR 1.41 [95% CI 1.29 to 1.54]) were associated with higher depression scores in comparison to men. Participants above the age of 60 years (RR 1.26 [95% CI 1.12 to 1.42]) were associated with higher levels of depression in comparison to 18–39 and 40–50 years olds. Participants who received no education were associated with higher depression in comparison to those that attended primary education (RR 0.80 [95% CI 0.71 to 0.90]) and those that attended higher than elementary (RR 0.79 [95% CI 0.71 to 0.87]). Participants presenting with leprosy, (RR 1.44 [95% CI 1.26 to 1.65]), Buruli ulcer (RR 1.45 [95% CI 1.21 to 1.73]) and LF-related lymphoedema (RR 1.90 [95% CI 1.67 to 2.17]) were associated with higher depression scores in comparison to LF-related hydrocoele. Participants with a mild anxiety (RR 2.74 [95% CI 2.38 to 3.16]), moderate anxiety (RR 3.99 [95% CI 3.47 to 4.58]), severe anxiety (RR 5.05 [95% CI 4.26 to 5.99]), were associated with higher levels of depression in comparison to no anxiety. Participants with a mild disability (RR 2.20 [95% CI 1.69 to 2.87]), moderate anxiety (RR 4.15 [95% CI 3.19 to 5.41]), or severe anxiety (RR 4.75 [95% CI 3.57 to 6.31]), were associated with higher levels of depression in comparison to no disability.

**Figure 4:**
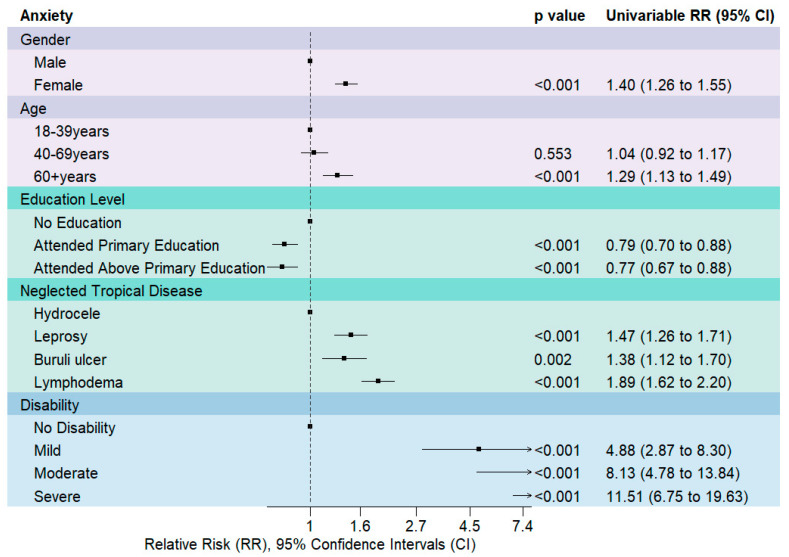
Relative-risk Ratios (RR) and 95% CIs for anxiety derived from univariable generalised linear regression Poisson models. RR above 1 (dotted line) shows a greater association with anxiety and RR below 1 means a lesser association in comparison to the first item listed for each variable.

For anxiety, Figure 4 shows estimated RRs, 95% CIs and *p*-values for univariable generalised linear regression Poisson analysis with all candidate risk factors. Participants that were female (RR 1.40 [95% CI 1.26 to 1.55]) were associated with higher anxiety scores in comparison to men. Participants above the age of 60 years (RR 1.29 [95% CI 1.13 to 1.49]) were associated with higher levels of anxiety scores in comparison to 18–39 and 40–69 years old. Participants that received no education were associated with higher levels of anxiety in comparison to those that attended elementary (RR 0.77 [95% CI 0.67 to 0.88]) and those that attended higher than elementary (RR 0.79 [95% CI 0.70 to 0.88]). Participants presenting with leprosy, (RR 1.47 [95% CI 1.26 to 1.71]), Buruli ulcer (RR 1.38 [95% CI 1.12 to 1.70]) and LF-related lymphoedema (RR 1.89 [95% CI 1.62 to 2.20]) were associated with higher anxiety scores in comparison to LF-related hydrocoele. Participants with a mild disability (RR 2.12 [95% CI 1.56 to 2.88]), moderate anxiety (RR 4.02 [95% CI 2.96 to 5.46]), severe anxiety (RR 5.91 [95% CI 4.28 to 8.15]), were associated with higher levels of anxiety in comparison to no disability.

Key risk factors, gender and disability were identified respectively from gender-disaggregated data analysis (Table 1), univariable risk factor analysis (Figure 1 and Figure 3), multi-variable analysis, previous studies, and qualitative findings. Four multivariable models were performed, two models for each outcome variable: depression and anxiety, model 1 displayed no interaction between gender and disability, and model 2 an interaction, as described in Table 2. Across all models, we identified that higher disability scores were associated with an increased incidence risk ratio of high depression and anxiety scores, see Table 2.

**Figure 5:**
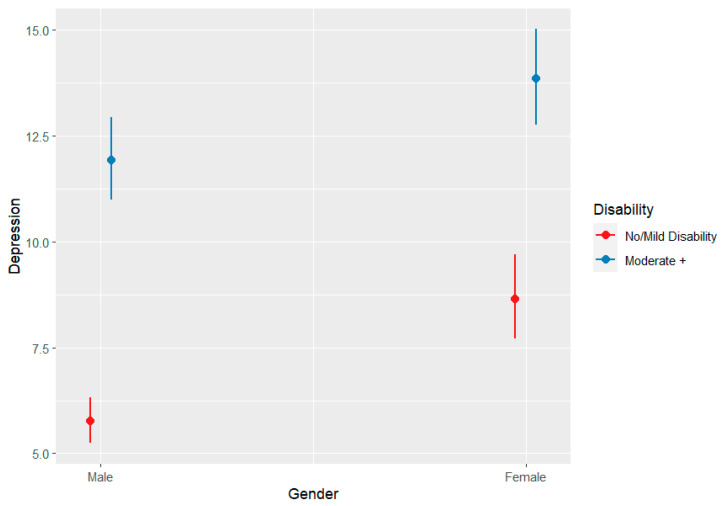
Interaction effect between gender and disability for depression.

We found that the incidence risk ratio for depression in females was higher in comparison to men, which was statistically significant across both depression models. For persons affected by NTDs, being a male and having no/mild disability moderated the negative effects of depression, as shown in Figure 5. The interaction between gender and disability in anxiety model 2 and being female in anxiety model 1 were not statistically significantly associated with a higher incidence risk ratio of anxiety. This shows for anxiety, disability has the greatest contribution to incidence risk ratio.

In the subsequent sections, we present further qualitative evidence linked to the differing domains of Meyer’s Minority Stress Model (2003), as adapted by Dean (2022) (see Figure 1), to consider the biosocial factors shaping this syndemic outcome. 

### 2.10. General Stressors

Our evidence shows the impact of general stressors for people affected by skin NTDs in Liberia. Specifically, they emphasise the influence of income losses and challenges with health seeking, largely due to accessibility related issues. 

### 2.11. Loss of Income Due to Physical Consequences of Their Condition

Physical limitations associated with skin NTDs were linked to financial difficulties due to loss of work, with some participants becoming dependent on relatives or begging as a means of survival. The perception of inappropriate appearance due to physical symptoms also posed challenges to livelihood activities with one person affected by leprosy describing being unable to wear shoes due to ulcers on his feet which created a barrier to office work. Some participants also described stigma (see below) leading to some people refusing to engage with them in selling or employment. Self-care, as a key strategy to managing skin NTDs, was described by some persons affected as important, however the time taken to do these activities was often outweighed by a need to provide for family members and go out to “look for my daily bread.” IDI with Male person affected by leprosy, aged 26 years, Lofa county.

*“…here most of my friends, some encourage me to work with them. [To] do letter work, pen and paperwork, but then, the problem that I have now, I can’t wear shoes.”*

***IDI with male person affected by leprosy, aged 26 years, Lofa***

*“…I learned the tailoring, I learned the tie and dye I can do some work, but only this my hand I do it with and this my hand can’t permit me.” *

***Bodymapping with female person affected by leprosy, Bong***

Both men and women described these negative livelihood impacts. However, adult men (aged 25–49 years) more commonly described both the economic consequences of their condition, as well as the negative impact of this on their own wellbeing. They frequently described feeling ‘bad’, worried, discouraged or anxious at being unable to go out and work to provide for their families. Some elderly participants described changing what they ate to cheaper alternatives due to lack of money.

*“I said the place I am now I am still feeling bad! I feeling bad of it, yes! Young man like me and this foot just get on me [pain in foot from NTD], I can’t be in peace, yes. I am still feeling bad about it and I am just asking God how we will be free from it.”*

***Bodymapping with male person affected, Grand Gedeh***

### 2.12. Health Seeking Challenges from Both Formal and Informal Providers

Accessing appropriate healthcare was identified as a challenge, driven by three key factors: (1) high costs associated with travel and lost work; (2) fear of medical procedures (e.g., amputation) within biomedical facilities; and (3) loss of confidence in the health system, largely due to irregular or absent medicine supply. These challenges often led to treatment cessation, as well as creating complex care pathways. For example, some have sought care from an informal provider followed by biomedical care, others vice versa, and some seeking care from both simultaneously. Several participants with lymphoedema described having had their foot *“cut open”* by a herbalist/traditional healer. Other traditional practices such as providing something to drink leading to patients experiencing vomiting and diarrhoea, feeling “helpless” as a result. This resulted in negative impact for the person affected, who felt like the herbalist was “trying to destroy them”.

*“Somebody [traditional healer] cutting human being flesh putting it down and say that’s medicine … He wanted to destroy me, so that’s the bad care people there.”*

***IDI with female person affected by lymphoedema, aged over 49, Margibi***

*“They say they don’t have the drugs there in the hospital. So, when we go there, they can only prescribe drugs and give it to us. Then we go buy the drugs in the other clinics. So, what is the use of [me] going there still?”*

***IDI with male person affected by Buruli ulcer, aged 69, Grand Gedeh***

Chaotic and complex care seeking pathways were largely responsible for people affected having ‘lost-peace’, ‘thinking too much’ and experiencing anxiety. This was particularly common for male participants around the time of their diagnosis, due to their limited knowledge about what to expect, including what treatment would be needed. Where participants had been on such complex care seeking journeys but were still searching for a cure, a sense of hopelessness and resignation was often articulated.

*“You know as a patient sometime when you sick, you can be discouraged. Sometime in the night you can’t sleep.”*

***IDI with male person affected by leprosy, aged 34, Lofa***

*“Starting from this year I have gone nowhere yet because I am tired now. I am just spending money and no result so, I am just tired sitting down now and waiting for God’s time.”*

***IDI with Female person affected by Buruli ulcer, aged over 49, Margibi***

### 2.13. External Minority Stressors: Experienced Stigma

Experienced stigma was commonly described by participants across counties, ages and genders and was largely attributed to: community members’ perception that the condition is transmissible by physical contact/proximity; smell coming from wounds; belief systems including superstition and witchcraft. Experienced stigma was described by participants with all conditions; persons affected by leprosy described it most frequently and extensively. One of the most frequent forms described by people with leprosy and lymphoedema was name calling, such as *“the old man that gets leprosy*” IDI with a female person affected by leprosy, aged 98, Grand Gedeh or *“rotten man”* IDI with a male person affected by lymphoedema, aged 25–49 years, Margibi. Other forms of stigma described include verbal abuse, mocking, being the subject of gossip within the community, having people spitting due to bad smell from an ulcer and having been driven away or isolated by others, including being carried to spend time in the ‘sick bush’. 

“*I even left the place. I left them because of the stigma*” Social mapping with male participants, Lofa

Participants often described ‘feeling bad’ or ashamed in response. The sense of isolation was described more frequently by female participants, with male participants describing continued support from their wife (although they had also experienced loss of friendships). However, one male participant described having experienced rejection from his family, which provoked an angry response.

*“... when you find yourself like this when you were not like that, family turned their back, friends turned their back... When you are well, you are for people. People will like you, they will come around you, you will do things together, but this type of condition now nobody can come to your rescue.”*

***Bodymapping with female person affected by Lymphoedema, aged 50, Margibi***

*“I am angry with my family... They cannot [do not] visit me, they cannot [do not] keep time with me... So, I am very angry of that. From there, when I sit down on my own when I think about my condition, I always cry, ‘what I do to my family they do not look at me, what I do to them they do not look at me.’”*

***Bodymapping with male person affected by Buruli ulcer, aged 47, Margibi***

Some participants also described experiencing discrimination from health workers if they showed any evidence of previously having sought care from an informal provider. One older female participant with lymphoedema gave a compelling story of a health worker refusing to treat her, shouting at her and ordering her to leave the facility. This stigmatising response led her to feel confused, adding additional stress for her when she was already feeling unwell.

*“They [name of hospital] didn’t even touch me! Later, when the Doctor came, he said “they touched this foot oh, because I can see chalk on it” so he didn’t even touch me. He didn’t feel the skin like this, he didn’t touch me; they just walked in group and went...and myself too I am sick and feeling bad and then I go somebody said “your carry her on that side, we don’t have place for her!” they were just shouting.”*

***IDI with female person affected by lymphoedema, aged over 49, Margibi***

### 2.14. Internal Minority Stressors

#### 2.14.1. Physical Symptoms

**Figure 6:**
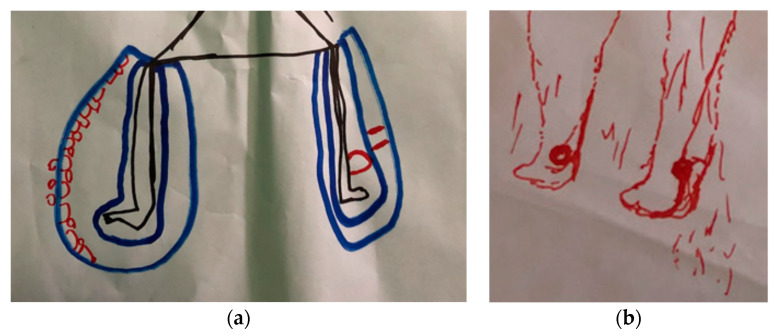
(**a**) Bodymap with male participant with Buruli ulcer, 69 years old, Lofa (**left**). (**b**) Bodymap with female participants, Margibi (**right**).

Participants described a wide range of symptoms related to their condition, including pain, swollen and/or itchy limb, ulcer/ sore, leaking ulcer, smell from ulcer, loss of sensation. These symptoms were frequently linked with a sense of shame and self-isolation by participants, particularly the foul-smelling exudate described by many participants with Buruli ulcer. The pain in particular impacted mental wellbeing among persons affected, contributing towards sleeplessness, loss of appetite and sadness. Bodymapping participants frequently showed pain, using red to indicate the severity of their pain linked to swelling and ulcers (Figure 6a,b). Other less commonly described symptoms include rash, loss of appetite, irritation from flies and several women described having lost their eyebrows, which they found distressing. 

#### 2.14.2. Internalised or Anticipated Stigma

People affected experienced shame related to physical symptoms, leading them to isolate themselves from others in the community, avoid social gatherings, with a reluctance to name or disclose their health condition, and describing themselves as ‘rotten’. It was common for participants to describe altering their clothing in order to try and hide their condition. These feelings were often described more intensely by younger participants.

*“Like for [me], [I] felt bad because looking at [my] age, [I am] very young and then, [I] get that kind of condition, [I] felt bad. Seeing [my] friends walking good, normal, and [I] have that condition, [I] felt very bad.”*

***Bodymapping with female person affected by lymphoedema, aged 30, Lofa***

#### 2.14.3. Coping and Social Support

Support from family, friends and faith in God were all described as forming essential coping strategies among persons affected. Participants regularly described placing their faith in God for healing, at times this was in response to having been abandoned by others. Faith was also described as playing a part in some people affected accepting their condition as being the will of God.

*“for me, I never lose hope because God is there, I never disappointed because the lord is always there for me.’’*

***Social mapping with male participants, Margibi***

*“I [am] sick, I not have nobody to help me only God. My children…they not big…Ain’t got no husband.”*

***Bodymapping with female person affected by lymphoedema, aged 50, Margibi***

Having someone to talk to was widely discussed as an important source of encouragement and companionship with a positive influence on overall wellbeing. For some, this role was played by the health worker, while for others if was a family member or friend. For those who were older, this support often came from children. In contrast among youth, aged 18–25 years, support was often came from their parents. Where support was present, this was highly encouraging for persons affected.

*“Yes, my family really catered to me in my sickness and they never gave me any word to make me feel bad, they make me always happy, and my community people, too, they did the same.”*

***Social mapping with male persons affected, Margibi***

The support included practical support such as helping to bring them to the health facility or to a traditional healer, as well as providing encouragement and emotional support. Where participants were married, gender was a clear mediator in shaping experience, with men often describing being supported by a spouse, whereas women frequently described being abandoned or alone. Male participants who received caring support from their spouse recognised the impact on their spouse.

*“When you find yourself like this when you were not like that, family turned their back, friends turned their back…When you are well, you are for people. People will like you, they will come around you, your will do things together, but this type of condition now nobody can come to your rescue.”*

***Bodymapping with female person affected by lymphoedema, aged 50, Margibi***

*“Lying down whole day and whole night, crying, the woman [wife] will come haul the clothes from under my foot and put different one there. She goes wash, hang it and put different one there. Five minutes, she comes again take that one, put different one there. Go wash it.”*

***Bodymapping with male person affected by Buruli ulcer, aged 41, Lofa***

Some participants felt that their friends continued to support them, after the diagnosis, although this was not universal and even those who generally felt supported by friends often still described instances of having been stigmatised by others in their community.

*“…some of my friends actually we move together, we do things together. Yes, they make me feel like them. they make me happy. They make me laugh. We all joke together, we do things together, we live happy life, but criticize. Some they stigma, some they talked about it, some even if you go around them, they take you different.” *

***IDI with male person affected by leprosy, aged 26, Lofa***

*“That’s just God, for me God love me and people been helping me, people know me, better people.”*

***IDI with female person affected by lymphoedema, aged over 49, Margibi***

## 3. Discussion

To our knowledge, this is the first study to presents mixed-methods approaches to evidence the syndemic relationship between NTDs and mental distress, specifically mental health conditions (depression and anxiety). Our study provides needed empirical evidence for the syndemic of concern, within the context of structural violence in Liberia, a post-conflict setting, with high levels of poverty, gendered social norms, stigma and weak health infrastructure [3]. This study used an adapted Meyers (2003) minority stress model, as is evidenced within other skin-NTD work [53,54,55], to present quantitative findings together with qualitative and participatory findings. Quantitative and qualitative findings informed analysis approaches to strengthen understanding in intersectionality for individual identities, stressors and syndemic outcomes mental health and disability (Figure 1). We utilised mixed-methods evidence in this under-researched area to support the collaborative design of holistic person-centred approaches to end syndemic suffering. The findings presented in this study were used to support intervention development workshops linked to the REDRESS programme, with findings presented in collaborative design workshops to shape recommendations for intervention design. Specifically, we illuminate the need for early case detection to prevent NTD-related disability and mental distress, as well as social and structural interventions to improve the health and wellbeing of those affected. Box 1 presents some of the recommendations for intervention areas identified following the presentation of these study results and that went on to be piloted within the REDRESS programme (results forthcoming).

Box 1 **Co-designed interventions developed in response to findings.**

- Delivery of awareness about NTDs and mental health in marketplaces and on the radio, led by community advisory boards that constitute of multiple community stakeholders.
- Establish peer support groups or networks for persons affected by skin NTDs to provide opportunities for experience sharing and mutual support, with seed funding to support financial sustainability.
- Support community health workers and informal providers to recognise the signs and symptoms of NTDs and mental distress, including tackling myths and misconceptions to support early identification and referral.
- Support health–workers through the provision of training and resource materials to diagnose NTDs and provide support for mental distress, including enhancing communication skills and supporting stigma reduction.
- Integrate screening tools for depression (PHQ9) and anxiety (GAD7) at point of NTD diagnosis.
- Provide mhGAP training for selected health workers at facility level, highlighting mental health and NTD links.

### 3.1. The Syndemic Spiral

**Figure 7:**
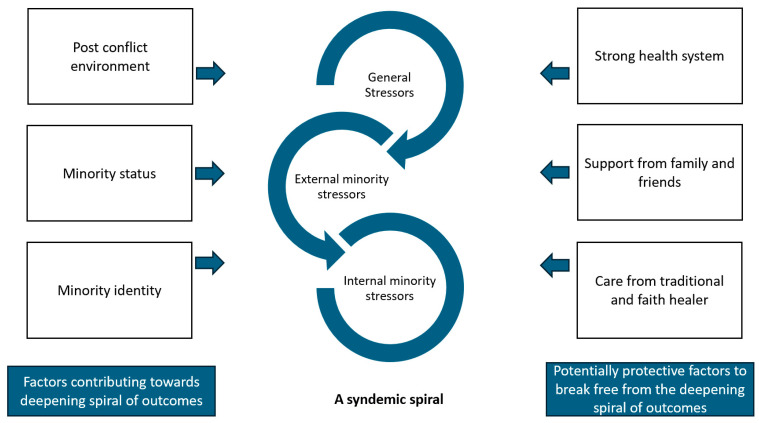
The Syndemic Spiral of NTDs and Mental Distress in Liberia.

Our findings align with those previously presented by Dean (2022) which emphasise that the post-conflict environment, minority status and minority identity of persons affected by NTDs interact with and exacerbate a person’s experience of generalised stressors. Their experience of external minority stressors, experienced through enacted stigma, and internal minority stressors, including physical symptoms and anticipated symptoms interact in a potentially ‘vicious spiral’ contributing to ever worsening physical and mental wellbeing. These effects can be buffered (or exacerbated) through the presence (or absence) of a strong health system, robust support from family and friends and holistic, non-stigmatising care from trained and trusted faith and traditional healers (Figure 7). 

### 3.2. General Stressors

In our study, we found one of the main general stressors described by persons affected was their experience of financial insecurities in consequence of their frequent loss of work due to the physical impacts of their condition. Similar to results from Nigeria where a contextually adapted collaborative care model for persons affected by skin-NTDs was developed for implementation in the primary care system [56]. Thus, the provision of economic support to persons affected is a key potential strategy to address the ‘syndemic spiral’. This could include the promotion of income generation activities for persons with disabilities, such as socio-economic rehabilitation, i.e., training in skills, educational opportunities and enhancing entrepreneurship [57,58,59]. Understanding the best and most sustainable ways to provide this economic support is likely to rely on linkages across sectors to ensure a person-centred response [17].

Alongside the financial insecurities, challenges related to health seeking via both formal and informal providers stemmed from pursuit of a ‘cure’, widely described in the literature, related to underlying health beliefs about causation of the condition, as well as health system limitations [60]. This oscillation between providers and inability to understand the cause of their condition has previously been described as distressing for persons affected [3,31]. Given the reliance of persons affected by NTDs on informal providers (both traditional and faith healers), as well as the holistic nature of care provided for persons affected by these informal providers in Liberia [61], there is opportunity to engage with traditional and faith healers, providing them training and mentorship to identify and refer persons affected, while supporting them to provide basic psychosocial support and referral (Box 1). Fear of biomedical care and frequent drug stock-outs created a loss of confidence in seeking care from the formal health system, as previously documented [62]. Thus, a strong supply chain has been described as a cornerstone for integrated case management [62]. 

### 3.3. External Minority Stressors

Enacted stigma was widely described by participants in the form of name calling, mocking, verbal abuse, being driven away by others, including being carried to the ‘sick bush’ [out of the community into the forest] which has previously been described in other studies among persons with NTDs in Liberia [63]. Quantitative findings emphasise the association between stigma with depression and anxiety. The experience of stigma by persons affected by skin NTDs has been widely described in the literature across multiple contexts and settings [64,65,66]. In our study, the experience of stigma was linked and often catalytic of mental distress, related to a sense of shame, isolation and loneliness from having been rejected. Social stigma related to disease has been identified as a key promotor of syndemic interactions and overall suffering [67], previously identified in the stigma syndemic of mental distress and LF/NTDs (Malawi and Liberia respectively) [3,57]. Stigma can further complicate recognition, treatment and prevention of disease [67]. Links between stigma, mental health and NTDs are well documented [15,64]. Challenging stigma through the work of peer support groups, and training of local community leaders and change agents such as traditional and faith healers were recommended as necessary strategies to respond to some of these findings (Box 1). Peer support groups have been shown to improve wellbeing and self-esteem amongst persons affected by skin-NTDs, as well as can encourage adherence to self-care [68]. Wider community stigma reduction interventions such as community awareness campaigns, stepping stones, and community conversations, have been implemented in other settings and relevant to other infectious diseases to support more holistic responses to the stigma experienced (HIV in South Africa and NTD podoconiosis in Ethiopia) [69].

### 3.4. Internal Minority Stressors

Physical symptoms caused participants’ considerable distress as described in the qualitative findings, and from the quantitative findings, greater degrees of disability were associated with higher levels of depression and anxiety. Experience of pain relating to Buruli ulcer and lymphoedema has previously been widely described [70,71], with up to 95% of persons affected by Buruli ulcer describing pain in one study from Ghana [71]. Alongside pain, symptoms of anxiety contributed to further physical symptoms, such as the inability to sleep and loss of appetite. The interlinking of symptoms between NTDs and mental health, creates a vicious cycle which can self-perpetuate with pain contributing to sleeplessness, contributing to anxiety and depression, contributing to reduced appetite, potentially contributing to worsening of physical symptoms related to poor nutrition. 

Other physical symptoms such as foul-smelling exudate and leaking wounds among persons affected by Buruli ulcer contributed to feelings of shame and internalised and/or anticipated stigma among persons affected, leading them to isolate themselves from others within their community. In this study, surveys showed women to have statistically higher internalised stigma scores in comparison to men. The enacted, anticipated and internalised stigma experienced by persons affected by Buruli ulcer and lymphoedema is in keeping with findings from a systematic review exploring social stigma towards NTDs [64]. 

### 3.5. The Equity Implications of NTD and Mental Health Syndemic Relationship

The existing structural and social inequities resulting from the macro-forces shaping Liberia’s complex history and present situation (including war, high poverty and low educational levels), mean the people affected by skin NTDs are frequently already among those most marginalised within communities at the onset of their symptoms. Experiencing the added generalised (financial concerns), external (experience of stigma) and internal (experience of pain and physical symptoms) minority stressors which persons affected in our study describe (to varying extents) contributes towards mental distress and the development of mental health conditions. Related to this, persons affected by NTDs and mental health conditions experience a loss of power, and restriction in participation in the life of their community and/or family, emphasised in a recent review of mental health, stigma and NTDs [15]. At times, this may be self-imposed due to anticipated stigma, or imposed by the community, through enacted stigma, shaped by fear of infection, discrimination and ableism norms. By adopting an intersectionality approach within our analysis [72], we can see that the experiences of persons affected are not homogenous, rather, there are many variations shaped by the unique circumstances of power, privilege and identity experienced in the life of each individual that shape the underlying pathways toward syndemic suffering. We found gender, age, (dis)ability and socio-economic status to be important ‘axes of privilege, power and inequity’ [73] shaping this path. 

### 3.6. Addressing Gender Inequities

This work further evidences the need to address gender-based inequalities and disparities within NTD and mental health programmes [74]. This gendered impact of NTDs creates a disproportionate burden on women and girls, largely due to gender roles and responsibilities, increased experience of stigma, discrimination and lack of spousal support; resulting in social and financial losses for women [75]. This disproportionate effect of the social consequences of NTDs for women, may explain the higher co-occurrence of depression and anxiety among women, in our study. Meanwhile, men of working age in our study described greater distress relating to their loss of ability to work due to their condition, and their inability to fulfil the traditionally male breadwinner role. This may have been shaped by the existing “hypermasculinity” norms previously described within Liberia [76] but warrants further exploration in subsequent work. Younger persons affected experienced distress at the changes they experienced in their life circumstances (loss of educational and work opportunities, and of ‘good health’), which they considered to be unfair at their stage of life [57]. Transforming the social, environmental and political factors which contribute towards the interactions between health conditions is needed to reduce the burden of ill health associated with NTDs and mental distress for all [3]. 

### 3.7. Limitations and Trustworthiness

This study benefited from mixed-method approaches where qualitative findings helped to interpret and add meaning to quantitative results. Some of the qualitative data were collected at different time points and in different counties; however, the findings were consistent, adding an additional layer of triangulation to the data. The role of co-researchers (including community health workers and a persons affected) as part of the research cycle, including data collection and co-analysis, provided opportunity to strengthen the trustworthiness of this work. The absence of a control group measuring depression and anxiety of the general population, does not allow us to make comparisons to overall population in Liberia, and quantitative prevalence studies are somewhat limited in Liberia [30]. The missing data entries from SARI stigma scores should be acknowledged when interpretating quantitative stigma findings presented in this paper. There is limited evidence on the validity, cultural interpretability and the use of the PHQ-9 and GAD-7 in Liberia, which may introduce measurement bias, and possibly underestimate the prevalence of mental health outcomes in this survey study group. 

## 4. Conclusions

We find strong evidence which adds to our understanding of the syndemic relationship between NTDs, and mental distress (anxiety and depression) within the context of structural violence in Liberia, in response to generalised, external minority and internal minority stressors. Persons affected by NTDs currently rely upon two main coping mechanisms, centred around support from their faith in God, and emotional, financial and social support from family and/ or friends. NTD programmes should strengthen these naturally occurring coping strategies, by providing training for faith healers, family and friends to provide basic psychosocial support, as well as strengthening multi-sectoral collaboration to support persons affected with opportunities for suitable income-generation activities. 

## Figures and Tables

**Table 1:** Gender-disaggregated analysis and illustrative qualitative evidence.

	Total, N (%)	Women, N (%)	Men, N (%)	Comparison	Illustrative Qualitative Evidence
Total	201 (100)	76 (37.8)	125 (62.1)		
***Depression***					
PHQ-9 mean SD	9.4 (6.1)	11.5 (5.8)	8.2 (6.0)	(t(164) = 3.92, *p* < 0.001) *	*“Very bad, I couldn’t sleep at all and I couldn’t eat because I only drank water for about one month, two weeks, I only live on water.” **Bodymapping with male participant, Grand Gedeh*** *“I was feeling bad and crying and don’t know what to do.” **Bodymapping with male participant, Grand Gedeh***
***Depression Clinical Scores***					
With depression (PHQ-9 ≥ 10)	96 (47.8)	46 (60.5)	50 (40.0)		
None (0–4)	49 (24.4)	10 (13.2)	39 (31.2)	X^2^ = 37.831, df = 4, *p* < 0.001 *	
Mild (5–9)	56 (27.9)	20 (26.3)	36 (28.8)		
Moderate (10–14)	56 (27.9)	23 (30.3)	33 (26.4)		
Moderately severe (15–19)	28 (13.9)	16 (21.1)	12 (9.6)		
Severe (20–27)	12 (6.0)	7 (9.2)	5 (4.0)		
***Self-harm/Suicidality***	96 (47.8)	50 (65.8)	46 (36.8)	(t(173) = 2.68, *p* = 0.008) *	*“I was feeling bad myself and even [at] that time I was having bad plan about myself for me to kill myself because I was feeling too bad. So, I say let me just die one time. It’s not good living here inside.” **Bodymapping with male participant with Leprosy, Nimba***
***Anxiety***					
GAD-7 mean, SD	7.1 (4.9)	8.6 (5.0)	6.1 (4.6)	(t(164) = 3.94, *p* < 0.001) *	*“Sometimes I can be thinking because if I sit down sometimes, I can be thinking too much, especially this sickness business.’’ **Social mapping with male participant, Margibi*** *“You know as a patient sometime when you sick, you can be discouraged. Sometime in the night sleep all you can’t sleep.” **IDI with male participant with hydroocele, aged 34, Lofa***
***Anxiety Clinical Scores***					
Moderate anxiety or above (GAD-7 ≥ 10)	62 (30.8)	34 (44.7)	28 (22.4)		
None (0–4)	74 (36.8)	21 (27.6)	53 (42.4)	X^2^ = 11.462, df = 3, *p* = 0.009 *	
Mild (5–9)	65 (32.3)	21 (27.6)	44 (35.2)		
Moderate (10–14)	48 (23.9)	27 (35.5)	21 (16.8)		
Severe ( >15)	14 (7.0)	7 (9.2)	7 (5.6)		
***Stigma***					
Total SARI mean, SD	22.6 (14.5)	25.8 (15.8)	20.5 (13.3)	(t(93.5) = 1.97, *p* = 0.051)	
SARI experienced mean, SD	7.5 (5.9)	8.6 (6.3)	6.9 (5.6)	(t(139) = 1.87, *p* = 0.064)	*“When it is on you most people don’t like to get around you; your friends themselves, some of them don’t like to be around you. So, I have been experiencing all those ones them, since this sickness got on me.” **IDI with male participant with hydrocoele, aged 73, Grand Gedeh***
SARI internalised mean, SD	5.7 (4.2)	6.8 (4.6)	4.9 (3.7)	(t(90.9) = 2.54, *p* = 0.013) *	*“The water was just coming out, myself, I used to be ashamed to go among my friend then. The water used to be so stink.” **Bodymapping with male participant with Buruli ulcer, aged 47, Lofa***
SARI anticipated mean, SD	4.1 (3.5)	4.7 (3.7)	3.6 (3.3)	(t(101) = 1.67, *p* = 0.097)	*“Because whenever I walked, they see my foot they start to laugh at me, so it makes me shy to go anywhere.” **Social mapping with male participant, Grand Gedeh***
***Disability***					
WHODAS mean SD	9.4 (6.1)	11.5 (5.8)	8.2 (6.0)	(t(158) = 3.64, *p* < 0.001) *	*“I can’t stand on my feet so because of that I am not able to walk to go sit down to the town hall.’’ **Social mapping with female participant, Lofa*** *“Yes, as for me, because of my foot business I cannot even go to the market.’’ **Social mapping with male participant, Grand Gedeh***
***Disability Category Scores***					
No disability	18 (8.9)	3 (3.9)	15 (12.0)	X^2^ = 10.752, df = 3, *p* = 0.013 *	
Mild	102 (50.7)	33 (43.4)	69 (55.2)		
Moderate	60 (29.9)	30 (39.5)	30 (24.0)		
Severe	16 (8.0)	9 (11.8)	7 (5.6)		

* Statistically significant *p* < 0.05 in paired *t*-test (t) and independence chi squared test (X^2^).

**Table 2:** Multivariable generalised linear regression Poisson models for depression and anxiety. Presenting an interaction (model 2) and no interaction (model 1) between gender and disability.

	Depression Model 1			Depression Model 2			Anxiety Model 1			Anxiety Model 2		
Characteristic	IRR ^1^	95% CI ^1^	*p*-Value	IRR ^1^	95% CI ^1^	*p*-Value	IRR ^1^	95% CI ^1^	*p*-Value	IRR ^1^	95% CI ^1^	*p*-Value
***Gender***												
Male	—	—		—	—		—	—		—	—	
Female	1.15	1.05, 1.27	0.003	2.00	1.51, 2.66	<0.001	1.11	0.99, 1.23	0.067	1.40	1.0, 1.97	0.052
***Disability***	1.03	1.03, 1.04	<0.001	1.04	1.04, 1.05	<0.001	1.04	1.04, 1.05	<0.001	1.04	1.04, 1.05	<0.001
Gender * Disability												
Female * Disability				0.98	0.97, 0.99	<0.001				0.99	0.98, 1.00	0.2
Log-likelihood	−669			−661			−555			−554		
AIC	1344			1330			1116			1116		

^1^ IRR = Incidence Rate Ratio, CI = Confidence Interval, * = interaction.

## Data Availability

All data generated and analyzed during this study are included in this manuscript. Raw qualitative data are not available and will not be publicly shared, as this would compromise the anonymity and protection of our study participants.

## Supplementary Materials

The following supporting information can be downloaded at: https://www.mdpi.com/article/10.3390/tropicalmed9080183/s1, Table S1: Means (SD) for each continuous outcome measure and proportion (N) for binary outcome by group for each socio-demographic characteristic. Table S2: Participant characteristics for qualitative and participatory research methods.
