# Supplementary material for: ‘The Lost Peace’: Evidencing the Syndemic Relationship between Neglected Tropical Diseases and Mental Distress in Liberia
Source: Trop Med Infect Dis. 2024 Aug 17;9(8):183. doi: 10.3390/tropicalmed9080183 (PMC11359536; doi:10.3390/tropicalmed9080183)
Supplement: Supplementary file 1 [file tropicalmed-09-00183-s001.zip › tropicalmed-3140339-supplementary.pdf]

Table S1. Means (SD) for each continuous outcome measure and proportion (N) for binary outcome by group for each socio-demographic characteristic.

Characteristics (Baseline)	Total, N (%)	Women, N (%)	Men, N (%)
Total	201 (100)	76 (37.8)	125 (62.2)
Neglected Tropical Diseases /clinical manifestations			
Buruli Ulcer	70 (34.8)	27 (35.5)	43 (34.4)
LF – Hydrocoele	49 (24.4)	NA	49 (39.2)
LF – Lymphoedema	54 (26.9)	36 (47.4)	18 (14.4)
Leprosy	21 (10.5)	11 (14.5)	10 (8.0)
Yaws	6 (3.0)	2 (2.6)	4 (3.2)
Onchocerciasis	1 (0.5)	NA	1 (0.8)
Age			
Mean, in years, SD	48.0 (15.7)	52.1 (16.4)	45.5 (14.7)
County			
Grand Gedeh	41 (20.4)	15 (19.7)	26 (20.8)
Lofa	73 (36.3)	19 (25.0)	54 (43.2)
Margibi	34 (216.9)	13 (17.1)	21 (16.8)
Grand Kru	53 (26.4)	29 (38.2)	24 (19.2)
Education Level			
No Education	78 (38.8)	48 (63.2)	29 (23.2)
Primary Education	44 (21.9)	13 (17.1)	31 (24.8)
Higher than Primary Education	79 (39.3)	14 (18.4)	65 (52.0)

Table S2. Participant characteristics for qualitative and participatory research methods.

Participant	Male				Female		
	18-25	26-49	50 plus	age unknown	18-25	26-49	50 plus
**In depth interviews**							
Person affected with leprosy		1(LO)					1(GG)
Person affected with lymphoedema		1(MG)					1(MG)
Person affected with hydrocoele		1(LO)	1(GG)				
Person affected with Buruli ulcer			1(GG)		1 (GG)		1(MG)
Person affected condition unknown	1(MG)			2(LO)			
**Body mapping**							
Person affected with leprosy	2(BO)	1(BO), 2 (NI)	2(NI)		1(LO)	2(LO)	1(BO)
Person affected with lymphoedema		1(MG), 1(BO), 1(NI)	1(BO), 1(NI), 3(GG)		1(LO)	2(LO), 1(MG), 1(GG)	1(BO), 1(GG)
Person affected with hydrocoele	1(MG)	2(GG)		1(LO)			
Person affected with Buruli ulcer	1(LO), 1(BO)	2(LO), 3(MG), 1(BO)	1(GG)	1 (LO)	1(MG), 1(NI), 3(GG)	2(MG), 1(BO), 3(NI), 1(GG)	1(LO), 1(BO), 1(GG)

Participant	Male				Female		
	18-25	26-49	50 plus	age unknown	18-25	26-49	50 plus
**Social mapping**							
Person affected male		3(MG), 2(GG)	2(MG, 4(GG)	3 (LO)			
Person affected female	1(GG)	2(MG), 1(GG)	2(MG), 3(GG)	5 (LO)			

LO=Lofa, GG=Grand Gedeh, MG=Margibi, BO=Bong, NI=Nimba
